# Keeping neurons in shape with fats: an educational primer for use with “Phospholipid biogenesis maintains neuronal integrity during aging and axon regeneration”

**DOI:** 10.1093/genetics/iyag061

**Published:** 2026-04-01

**Authors:** Ryan J Lee, John P Vaughen

**Affiliations:** Department of Anatomy, University of California San Francisco, San Francisco, CA 94143, United States; Department of Anatomy, University of California San Francisco, San Francisco, CA 94143, United States

**Keywords:** *C. elegans*, axon regeneration, lipid biosynthesis, education, WormBase

## Abstract

Neurons assemble into breathtakingly intricate circuits that govern behavior and often perdure for life. How injured or aged neurons control their complex morphological connections to survive or perish remains an exciting and open question. Using the genetics of *Caenorhabditis elegans*, Park et al. uncover that lipid biosynthesis is critical for both neuronal regrowth after injury, and for maintaining neuronal morphology during aging. This primer is designed to help students read and understand the paper, “*Phospholipid biogenesis maintains neuronal integrity during aging and axon regeneration*.” We emphasize the utility of model organisms, highlight the power of *C. elegans* neuroscience, provide background on lipids and neuron regeneration, and discuss genetic nomenclature and techniques. We then provide a set of questions for each figure in Park et al. designed to deepen students' reading of the data and text. Finally, we provide prompts to guide potential projects that students could conduct in an introductory genetics course to explore how these underappreciated fats underlie neuronal shape and function.

## The big picture

Neurons must maintain precise connections and shapes to sustain lifelong brain function. However, both brain injury and age can contribute to altered membrane morphologies, leading to severed and degenerated axons (Adams et al. 1989; Hou et al. 2019). Thus, understanding the molecular mechanisms of axon maintenance and regeneration may pave the way for the development of therapies to treat nervous system injuries or age-related neuronal damage. Importantly, lipids are major and integral parts of cellular membranes that define neuronal shapes. Thus, the continual biosynthesis, breakdown, and remodeling of lipids is intimately linked to neuronal morphology. Here, we review how Park *et al*. seek to understand how lipid metabolism controls neuronal morphology after axotomy, the severing of a neuron's axon, and during aging.

Park et al. find that without *cept-2* or *ept-1*, 2 enzymes critical for the biosynthesis of major membrane lipids, neuronal axons are unable to regenerate after injury. Moreover, these 2 enzymes are also required for maintaining neuronal morphology during aging, with CEPT-2 required cell-autonomously in specific mechanosensory neurons. They also find that the loss of *dip-2*, a poorly understood regulator of lipid metabolism, can rescue regeneration in *cept-2* or *ept-1* mutants. Taken together, this work highlights the importance of lipid metabolism for enduring injury or aging in the nervous system.

## Model organisms and *Caenorhabditis elegans*

“What is true for *E. coli* is true for the elephant” opined the Nobel Laureate Jacques Monod. Because genetics and the fundamental rules of molecular and cellular biology are well conserved across all species on Earth, studying simpler “model” organisms can reveal general principles of biology that illuminate cellular mechanisms pertinent to human physiology and disease. Common models include bacteria (*Escherichia coli*), mustard plants (*Arabidopsis thaliana*), baker's yeast (*Saccharomyces cerevisiae*), mice (*Mus musculus*), fruit flies (*Drosophila melanogaster*), zebrafish (*Danio rerio*), and worms (*Caenorhabditis elegans*). Over the past century, these and other model organisms have become an essential backbone of biological and biomedical research. Indeed, this year's Nobel Prize in Physiology or Medicine was awarded for autoimmune work conducted in mice. Over 2 decades ago, the 2002 prize was awarded to Sydney Brenner, H. Robert Horvitz, and John E. Sulston for landmark genetic discoveries of cell development and death using the worm model. “Without doubt the fourth winner of the Nobel prize this year is *Caenorhabditis elegans*; it deserves all of the honour but, of course, it will not be able to share the monetary award,” quipped Brenner in his Nobel lecture.

More than 50 yr ago, Sydney Brenner believed that understanding behavior would come through genetic advances that detailed how the nervous system was formed and then functioned. In his seminal 1974 *Genetics* paper, “The genetics of *Caenorhabditis elegans*,” he showcased his novel organism, *C. elegans*, identifying 77 mutations that altered animal movement (Brenner 1974). Since then, *C. elegans* has become one of the most popular models in biological research. This is for good reason: they are easy to handle and maintain (1 mm long, picked under microscopes with fine picks, fed on a small *E. coli* lawn), are self-fertilizing hermaphrodites that produce ∼300 offspring, can be genetically crossed, have a short reproductive life cycle (3.5 d), and are transparent. Moreover, each cell has an invariant fate, allowing precise mapping of cell birth, differentiation, and death throughout life. These traits enabled numerous pioneering studies: *C. elegans* was the first multicellular organism to have its full genome sequenced, each of its 959 somatic cells mapped to a precise developmental lineage, and its neuronal connectome elucidated (Corsi et al. 2015). As a result, genetic and molecular techniques for working with *C. elegans* are well-established, providing scientists with an incredible toolbox to precisely control genes and proteins in specific cells and life stages.

## *C. elegans* neurons

**Fig. 1.**
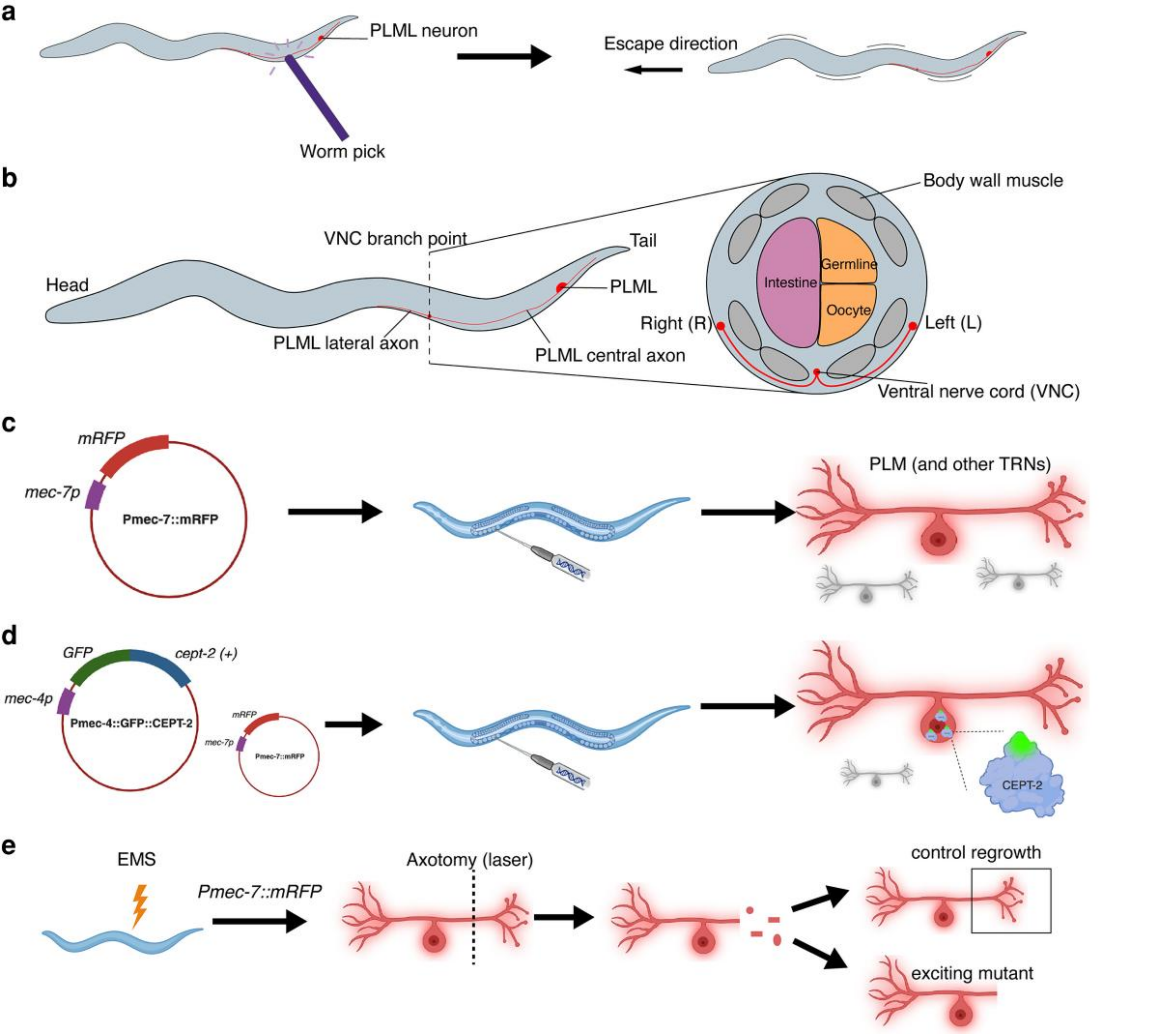
PLM localization, function, and genetic constructs used to study PLMs. a) When worms are lightly touched on the posterior, PLM neurons trigger an escape response. b) Model of *C. elegans* PLM neurons. Note bilateral symmetry with L (left) and R (right) PLM neurons. c) Diagram of plasmid injected to visualize TRNs including PLM using mRFP (red haze), and d) the plasmids injected to visualize GFP::CEPT-2 in a RFP + PLML; the protein structure of CEPT-2 is fictional, GFP (green) is not drawn to scale. Unlabeled neurons are depicted in gray. e) Schematic of a potential genetic screening approach to identify novel genes involved in axon regeneration via random EMS-induced mutations followed by PLM axotomy and then confocal imaging of neurons for altered patterns of regrowth (“exciting mutant”). Created in BioRender. Lee, R. (2026) https://BioRender.com/i84um3b.

*C. elegans* have 302 neurons that form ∼7,000 synaptic connections (WormWiring). Most of the neuronal soma (cell bodies) are clustered in the head's nerve ring, but soma also exist along the body axis and tail. In Park et al., the neurons of interest are touch receptor neurons (TRNs), which refer to 6 mechanosensory neurons (ALML/R, PLML/R, AVM, PVM) that respond to gentle touch, such as a fine hair brushing across the body of a worm (Fig. 1a). Park et al. study the PLML/R neurons, bilateral neurons located close to the worm's surface that are easily visualized, ablated, and tracked as they robustly regenerate a long anterior axon (Fig. 1b).

Park *et al*. use a fluorescent reporter to visualize both TRNs and the localization of CEPT-2 within these neurons. To visualize a specific neuronal cell-type such as TRNs, the DNA encoding the fluorescent label is preceded by a cell-type-specific promoter and its relevant regulatory elements. Thus, to visualize TRNs, Park et al. use a *mec-7* promoter, which selectively expresses in the 6 TRNs, combined with a mRFP construct (Fig. 1c). Using fluorescence microscopy, mRFP can be excited to produce red light for imaging, thereby illuminating the TRNs. Park et al. were also interested in identifying the subcellular localization of CEPT-2 in PLMs. Using *mec-4*, a different TRN promoter, they combined GFP and CEPT-2 encoding constructs (Fig. 1d). What would we expect to see? GFP fluorescence where the CEPT-2 protein is localized within TRNs.

## Studying axon regeneration

How injured neurons recover remains an outstanding and pressing question. Unlike many other cells in the body, such as skin or gut cells, most neurons perdure for life, meaning that a failure to regenerate prior connections can cause permanent defects in neuronal connectivity and behavior. While peripheral nervous system (PNS) axons have regenerative capacity, the neurons within the central nervous system (CNS) are refractory to regeneration in humans and many other species. Thus, determining the molecular and genetic mechanisms enabling or preventing axon regeneration could unlock therapies for reverting or allaying neurodegenerative diseases.

To study regeneration, an injury model is needed. The standard method for axonal injury is by laser ablation, where a high-powered laser precisely targets a single axon. The Chisholm lab previously conducted a genetic screen to discover genes involved in axon regeneration (Chen et al. 2011). Genetic screens are powerful because they identify new genes as critical players in the biological process being studied. In Chen et al., 654 genes were screened using both loss-of-function and gain-of-function mutations (see below section on genetics). After axons were severed by laser-ablation, confocal imaging 24 h post-injury determined regrowth capacity of controls vs these mutants (Fig. 1e). What phenotype is expected for genes crucial for axon regrowth? Loss-of-function mutations of these genes should block regrowth, implicating these genes as pro-regenerative. Conversely, loss-of-function mutations that cause excess regrowth would implicate genes that normally function to suppress axon regeneration. Novel hits from genetic screens can be characterized in follow-up studies. For example, Park et al. now focus on phospholipids, which were implicated in axon regrowth in both worms and mice (Kim et al. 2018; Yang et al. 2020).

## *C. elegans* genetics

One of the major advantages of working with *C. elegans* is the ease with which scientists can manipulate its genes. Classically, genetic experiments come in 2 main flavors: loss-of-function experiments, which reduce or completely block the function of the gene product, and gain-of-function experiments, which introduce a new function of the gene product, often by overexpressing the gene. By observing what happens behaviorally or physiologically, scientists can then deduce the normal function of the gene. Among loss-of-function experiments, there are knockout, knockdown, and pharmacological inhibition experiments. The complete removal of gene function generates a “null allele.” In *C. elegans*, gene knockouts were historically made broadly and blindly using a mutagen, such as ethyl methanesulfonate (EMS), to induce many DNA mutations, from which a mutant of interest could then be isolated. However, with the advent of gene manipulation techniques such as PCR, Gibson assembly, and CRISPR, scientists can now design, target, and change specific sequences of DNA in a highly controlled manner. For example, Park et al. use CRISPR to generate precise mutations in *cept-2* and *ept-1*.

These gene-editing tools, coupled with DNA synthesis, have also enabled us to design and produce exogenous DNA known as transgenes, which are genes that are added into an organism. Park et al. use minigenes and molecular tags, two of the most common transgene types. Minigenes are constructs containing the minimally required components of a gene, typically its exons and regulatory elements needed for wild-type expression. Minigenes are often used for rescue experiments and to explore how elements of genes regulate gene function. For example, Park et al. use a *cept-2* minigene to rescue the defects of *cept-2* null mutants. In contrast, molecular tags are often used for visualizing where a protein is found inside an organism or cell. These tags can be endogenously fluorescent, such as RFP or GFP, or antigens (ie FLAG, V5, myc, HA) that are then visualized by secondary fluorescent tags.

After generating a transgene, how do we express it in our model? For *C. elegans*, scientists microinject plasmids that contain the desired transgene. The injection of many copies that remain extrachromosomal are known as extrachromosomal arrays, but the DNA can also be integrated into the chromosomes using DNA editing techniques like CRISPR. While direct integration takes more experimental effort, it enables precise and reproducible control of transgene levels in target cells, while extrachromosomal arrays can vary in expression strength within and between worms.

## *C. elegans* genetic nomenclature

With the numerous ways in which *C. elegans* can be genetically modified, standardized nomenclature is used to remove ambiguity. Genetic loci are given a 3-letter name followed by a hyphen and number. The 3 letters are often related to the gene's function. For example, *ept-1* encodes the enzyme **E**thanolamine **P**hospho**T**ransferase 1. In mutant worms, mutant alleles are also given unique identifiers, a 2-letter name followed by a number. For wild-type alleles, a “+” is used, and shorthand for null alleles is “0”. For example, in *ept-1(tm3093), tm3093* is the mutant allele identifier, and in this case represents a null mutation. Using our shorthand, we can substitute *tm3093* with *0*, thus writing *ept-1(0)*.

For transgenes, the first 2 letters indicate the laboratory followed by either *Ex* for extrachromosomal or *Is* for integrated, and finally a number. This is often further followed by square brackets that include genotypic information. For example*, juEx1[ept-1(+)]* indicates that this transgene is from the Chisholm lab (*ju*), is extrachromosomal, and contains a wild-type *ept-1*. Sometimes, as Park et al. do, the laboratory identifier is dropped, and the notation becomes *Ex1[ept-1(+)]*. The genotype information included in the square brackets typically includes the promoter and insertion. The promoter is represented by “P” followed by the gene from which the promoter is derived. In many transgenes, a tag is inserted to label the protein product, and is indicated by the “::” symbol. As an example, *juEx7992[Pmec-4-GFP::cept-2 cDNA]* indicates that the expression of the transgene *juEx7992*, which has GFP fused to wild-type CEPT-2, is specifically expressed in cells that activate the *mec-4* promoter. That is to say, GFP-labeled CEPT-2 protein will be expressed in TRNs that express *mec-4* (Fig. 1c).

## Lipid homeostasis in neurons

**Fig. 2.**
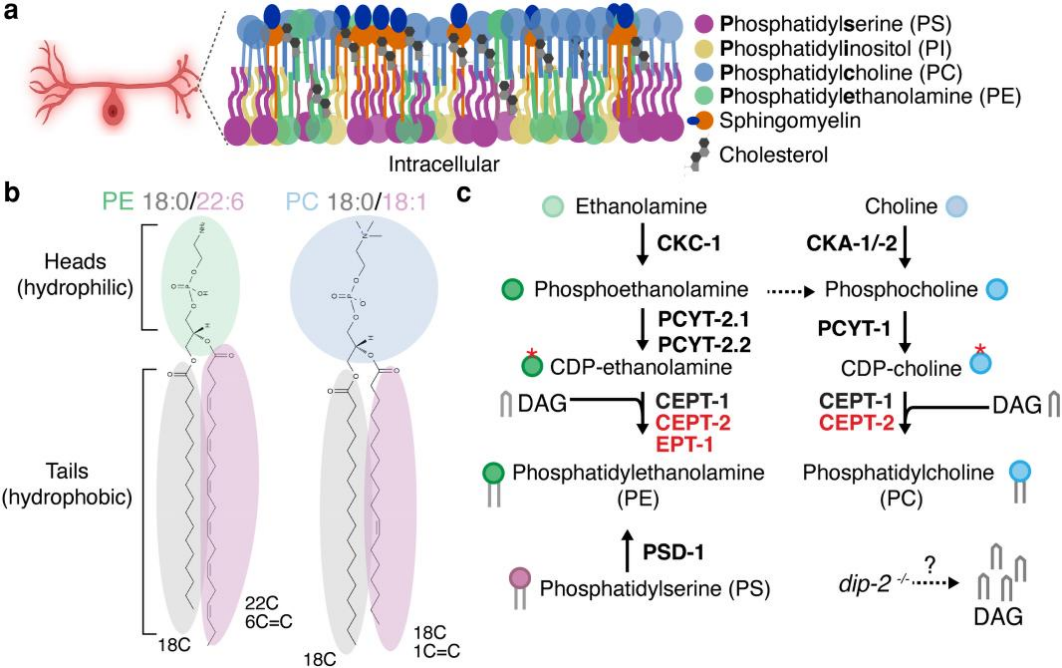
Membrane model, phospholipid composition, and synthesis of PE and PC. a) Model of neuronal plasma membrane containing phosphatidylserine (PS), phosphatidylinositol (PI), phosphatidylcholine (PC), phosphatidylethanolamine (PE), sphingolipids, and cholesterol; adapted from (Doktorova et al. 2025). b) Lipids contain diverse hydrophilic heads and hydrophobic tails, which differ in length and number of carbon–carbon double bonds. PE 18:0/22:6 and PC 18:0/18:1 are depicted; 18:0 refers to a tail with 18 carbons and no double bonds, while 22:6 refers to a tail with 22 carbons and 6 double bonds. c) the Kennedy pathway produces PE and PC starting from ethanolamine (green) and choline (blue). EPT-1 and CEPT-2 steps are highlighted. Mutations in dip-2 may increase DAG levels (grey brackets). Red asterisk indicates the CDP-activated phosphoethanolamine headgroup. Created in BioRender. Lee, R. (2026) https://BioRender.com/nrgbhc9.

What gives neurons their exquisite shapes and sizes? Their plasma membranes, which are composed of hundreds of diverse lipids (and proteins, too)! Lipids encompass a cornucopia of molecules that are amphipathic, meaning they have regions that are hydrophobic (water fearing) and other regions that are hydrophilic (water loving). For example, a prototypical lipid consists of a polar headgroup oriented toward water, with 2 fatty tails that hide from water in the hydrophobic core of the lipid bilayer (Fig. 2a, b). This unique property allows lipids to partition cells and organelles, thus enabling spatially localized biochemical reactions that are critical for functions ranging from neurotransmission via synaptic vesicles to plasma membrane expansion during wound healing. Within the lipid family, glycerophospholipids (glycerol backbone), sphingolipids (sphingosine backbone), and cholesterol constitute major components of most plasma membranes (Fig. 2a).

Park et al. focus on 2 major glycerophospholipids, phosphatidylcholine (PC) and phosphatidylethanolamine (PE). PE and PC can be synthesized via the Kennedy pathway in 3 enzymatic steps to convert choline and ethanolamine to PC and PE, respectively (Fig. 2c). Park et al. focus on *cept-2* (produces PC and PE) and *ept-1* (produces PE) (Gibellini and Smith 2010). While some lipids become stable end products with long half-lives, lipids often interconvert to other molecular species in a dynamic metabolic web. For instance, phosphatidylserine (PS) is converted into PE inside mitochondria (Fig. 2c). This fascinating feature of lipids makes direct inferences about how levels of lipid species change as a consequence of genetic perturbations challenging, especially as lipids vary extensively in abundance between cells, organelles, and even membrane leaflets.

## Experimental logic: figure questions

Students are encouraged to work through figures in groups, using the questions below as guides to deciphering the key points of each figure. The answer key is provided as a Supplementary file.

*Figure 1*. Is CEPT-2 required for injury-induced axon regeneration?

1. What step in the Kennedy pathway is CEPT-2 involved in?
2. What direct lipid changes are predicted from *cept-2* or *ept-1* mutations?
3. How do the authors normalize axon regrowth?
4. What kind of alleles are *ju1669* and *ok3135* and what are some similarities and differences between them?
5. Which genotype in Fig. 1d proves that *ju1669* defects are caused by loss of CEPT-2?

*Figure 2*. Is CEPT-2 important for normal maintenance of axons across age?

1. How are PLM neurons in *cept-2(0)* worms visualized? What makes *C. elegans* a particularly useful model organism for this visualization?
2. What does the *cept-2* minigene rescue show in Fig. 2a? If the *cept-2* minigene did not rescue, what would that imply about the observed phenotype?
3. What is the difference between *Pmec-7::mRFP* and *Pmec-4::GFP::CEPT-2*? What does the fluorescent signal from each transgene tell us?
4. Does CEPT-2 function autonomously or non-autonomously in aging neurons?
5. What might be inside the beading? How would you test this?

*Figure 3*. Is EPT-1 required for development and reproduction?

1. Is *C. elegans*  EPT-1 more similar to *C. elegans*  CEPT-2, or to human EPT-1?
2. How are *ept-1(0)* worms phenotypically distinct from wild-type worms? What assays were used to compare them?
3. List 2 reasons why the GFP::EPT-1 transgene may not fully rescue *ept-1(0)* defects.

*Figure 4*. Is EPT-1 required for axon regeneration or axon maintenance during aging?

1. How comparable are *ept-1(0)* axon phenotypes to *cept-2(0)* phenotypes for regeneration and aging? If there are differences, what lipid-based reasons could explain this?
2. Why did the authors want to test *ept-1(0); cept-2(0)* double mutants? Did they?
3. While the *ept-1* transgenes rescued proximal axon regrowth after axotomy, the authors noted that the terminal morphology of the neuron remained aberrant. What does it suggest about the different areas of axons, and the *ept-1* rescue constructs?

*Figure 5*. What is the role of DIP-2 in axon regrowth?

1. What do the single and double mutants (*dip-2(0); cept-2(0)* and *ept-1(ju1698)dip2(0)*) suggest about the function of DIP-2? Does DIP-2 function depend on PE biosynthesis?
2. Which condition had the most prominent mean axon regrowth (Fig. 5a, b)? What might this suggest about DIP-2 and DAG function in axon regrowth?
3. How might you test if the *dip-2* mediated regrowth was functional in the ALM circuit?
4. What other functions could lipids play in regrowth, outside of structural bilayer components such as PE/PC?
5. What phenotypes do authors notice occurring in the *dip2;cept-2* and *dip2;ept-1* double mutants? How do authors quantify these phenotypes?

## Future directions and experiments

1. DIP-2 prevented axon regrowth defects. How would you test if DIP-2 also protected against neurodegeneration in phospholipid biosynthesis mutants?
2. How could you use the approach from Park et al. to test how diet, a major source of lipids, influences axon regeneration?
3. You have probably heard that exercise and sleep are good for your brain. How would you test this in the context of axon regeneration during aging by using *C. elegans*?
4. You want to conduct a screen that identifies factors that enable CNS neurons to regenerate similarly to PNS neurons. How would you do this?
5. What behaviors or cell biological phenotypes would you want to screen for in *C. elegans*? How would you design the screen?

## Technical glossary

*Amphipathic* refers to the quintessential property of lipids: they are water-fearing (hydrophobic) and water-loving (hydrophilic).

*Brood size* refers to offspring number of the hermaphrodite parent worm; typically ∼250 to 300.

*Cell-autonomous* refers to intrinsic functions that act independently of the surrounding cells. These functions are self-determined. This is in contrast to *cell non-autonomous* which refers to functions that are influenced by extrinsic factors such as signals from other cells.

*CEPT-2* or diacylglycerol choline phosphotransferase catalyzes the conversion of cytidine-diphosphoethanolamine and cytidine-diphosphocholine to PE and PC by transferring the phosphoethanolamine and phosphocholine, respectively, to DAG.

*CNS* stands for the central nervous system, which is made up of the brain and spinal cord. The CNS processes and responds to the sensory information received from the PNS.

*DAG* stands for diacylglycerol, which is composed of 2 fatty acids attached to a glycerol.

*DIP-2,* or DISCO-interacting protein 2, is part of the DIP2 family of proteins important for the metabolism of diacylglycerol (DAG). They are proposed to regulate the conversion of a unique set of DAGs into triacylglycerols (TAGs), and act as inhibitors of injury-induced axon regeneration (Noblett et al. 2019; Mondal et al. 2022). In *C. elegans* neurons, DIP-2 localizes to the cytoplasm and regulates aging neuron morphology through adenylate-forming domains.

*Epistasis* refers to a genetic phenomenon where alleles of different genes interact in a non-additive fashion, with the phenotype of one gene masking the phenotype of another, or 2 genes synergizing to produce a novel phenotype distinct from a simple additive phenotype.

*EPT-1* or Ethanolamine Phosphotransferase 1 catalyzes the conversion of cytidine-diphosphoethanolamine to PE by transferring phosphoethanolamine to DAG. In mammalian cell culture this conversion occurs in the Golgi (Horibata et al. 2020).

EMS is a chemical mutagen for randomly introducing point mutations.

*Eutely* is the property of constant cell number. *C. elegans* are eutelic while humans are not.

*Extrachromosomal array* refers to a large DNA structure formed from the injection of artificial DNA. These arrays, as the name suggests, are extrachromosomal and can also be inherited (or lost) following mitotic divisions.

*Frame shift mutations* refers to mutations in DNA that shift the reading frame of the ribosome (i.e. nucleotide deletions that are not multiples of 3). Frameshift mutations are highly deleterious, leading to nonsensical polypeptides and often premature stop codons.

*Gene knockout* refers to the total inactivation of a gene. This is often done by deleting part or all of the gene using gene editing techniques like CRISPR.

*Gene overexpression* refers to the amplification of a gene. This is often done by injecting a large number of copies of the gene, or by using a stronger promoter than occurs endogenously.

*GOF* stands for gain of function and refers to a class of mutations that results in a new function for the gene's protein product. This is often the result of increased genetic expression that amplifies its normal function.

*Kennedy pathway* refers to producing glycerophospholipids in the endoplasmic reticulum via the *de novo* synthesis route for PE and PC from ethanolamine and choline, respectively.

*LOF* stands for loss of function and refers to a class of mutations characterized by the reduction or complete loss of function for a gene's protein product.

*Morphology* in biology refers to physical shape.

*Null alleles* are alleles that do not produce a functional gene product due to a null mutation.

*PE* stands for phosphatidylethanolamine and is a phospholipid that is found in cell membranes. It is made up of 2 fatty acids, a glycerol backbone, and an ethanolamine headgroup. In red blood cells, it is thought to reside on the inner leaflet of the plasma membrane (Lorent et al. 2020).

*PC* stands for phosphatidylcholine and is a phospholipid that is found in cell membranes. It is made up of 2 fatty acids, a glycerol backbone, and a choline headgroup. In mammals and *C. elegans*, it is the most abundant glycerophospholipid and is thought to mainly reside on the outer leaflet of the plasma membrane.

*PLM* stands for Posterior Lateral Mechanosensory neurons. They are a subtype of touch receptor neurons. PLMs are sensitive to light touch and have been implicated in memory and escape response (Pirri and Alkema 2012). They are complemented by the Anterior Lateral Mechanosensory (ALM) neurons in the anterior part of the worm.

*PNS* stands for the peripheral nervous system, which is made up of nerves that extend from and return to the brain and spinal cord. The PNS receives sensory input and relays signals to the CNS and has higher regenerative capacity than the CNS.

*TAG* stands for triacylglycerols, 3-legged lipids that are joined by a glycerol group. As a reservoir of lipid chains stored in lipid droplets, TAGs are a major source of cellular energy.

*Transgene* refers to any artificial DNA that is inserted into an organism. These are commonly used to study gene function because transgenes can be precisely engineered.

## Additional reading

*C. elegans*

Sydney Brenner’s 2002 Nobel Lecture
Review on CRISPR-based methods
Genetic screen experiment identifies genes that regulate programmed cell death
Discovery of the first microRNAs
Discovery of RNA interference (RNAi)
Discovery of the daf-2/IGF signaling pathway

Axon regeneration

*C. elegans* axon regeneration genetics
Intrinsic control of axon regeneration
Glia in regeneration

Lipids

Lipid diversity
Asymmetric lipids in plasma membrane
Lipid and carbohydrate metabolism in *Caenorhabditis elegans*

## Supplementary Material

iyag061_Supplementary_Data

## Supplemental Material

Supplemental material available at GENETICS online.
