# Supplementary material for: Keeping neurons in shape with fats: an educational primer for use with “Phospholipid biogenesis maintains neuronal integrity during aging and axon regeneration”
Source: Genetics. 2026 Apr 1;233(1):iyag061. doi: 10.1093/genetics/iyag061 (PMC13147525; doi:10.1093/genetics/iyag061)
Supplement: iyag061_Supplementary_Data [file iyag061_supplementary_data.docx]

**Answer Key to Lee and Vaughen 2026 Genetics educational primer for** “*Phospholipid biogenesis maintains neuronal integrity during aging and axon regeneration*”.

*Figure 1.* Is CEPT-2 required for injury-induced axon regeneration?

1. What step in the Kennedy pathway is CEPT-2 involved in?
   1. *Final step, to generate either PC or PE by condensing DAG with CDP-choline (making PC) or CDP-ethanolamine (making PE).*
2. What direct lipid changes are predicted from *cept-2* or *ept-1* mutations?
   1. *cept-2(0) mutants should lower both PE and PC, while ept-1(0) should lower PE.*
3. How do the authors normalize axon regrowth?
   1. *Divide by mean of control axon length after identical laser ablation and 24 hour regrowth period.*
4. What kind of alleles are *ju1669* and *ok3135* and what are some similarities and differences between them?
   1. *ju1669 deletes 4 nucleotides in exon 2, while ok3135 is a larger deletion through exons 3-5. Similarities: both are predicted to cause strong loss-of-function and early frame-shifts.*
5. Which genotype in Fig. 1d proves that *ju1669* defects are caused by loss of CEPT-2?
   1. *Genotype: Ex1[cept-2(+)]+ ju1669 (columns 4-5), in which the cept-2 minigene is used to re-express CEPT-2 in the ju1669 mutant.*

*Figure 2*. Is CEPT-2 important for normal maintenance of axons across age?

1. How are PLM neurons in *cept-2(0)* worms visualized? What makes *C. elegans* a particularly useful model organism for this visualization?
   1. *Pmec-7::mRFP worms express cytosolic mRFP in PLMs and Pmec-4::GFP expresses GFP in PLMs. C. elegans is transparent, allowing direct microscopic observation.*
2. What does the *cept-2* minigene rescue show in Fig. 2a? If the *cept-2* minigene did not rescue, what would that imply about the observed phenotype?
   1. *The rescue shows that the striking 100% beading phenotype observed only in aged worms is caused by the loss of CEPT-2. If the rescue did not reduce the beading phenotyping, then a different defect in the cept-2(0) line (an unrelated mutation) could have caused the beading phenotype.*
3. What is the difference between *Pmec-7::mRFP* and *Pmec-4::GFP::CEPT-2*? What does the fluorescent signal from each transgene tell us?
   1. *Pmec-7::mRFP labels PLM neuronal cytosol with mRFP, while Pmec-4::GFP::CEPT-2 expresses GFP::CEPT-2 inside these neurons.*
4. Does CEPT-2 function autonomously or non-autonomously in aging neurons?
   1. *Autonomously, as specific induction of CEPT-2 in PLMs (using Pmec-4::GFP::CEPT-2) fully rescued the beading defect (Fig. 2f).*
5. What might be inside the beading? How would you test this?
   1. *Trapped organelles, protein aggregates, ectopic membrane structures (among other possibilities!). To test, could visualize with electron microscopy of the beading, or fluorescent staining and confocal microscopy in aged cept-2 mutants.*

*Figure 3.* Is EPT-1 required for development and reproduction?

1. Is *C. elegans* EPT-1 more similar to *C. elegans* CEPT-2, or to human EPT-1?
   1. *More similar to human EPT-1 according to the phylogenetic tree in 3a.*
2. How are *ept-1(0)* worms phenotypically distinct from wild-type worms? What assays were used to compare them?
   1. *Mutants are shorter and produce smaller broods (body length and brood assays).*
3. List two reasons why the GFP::EPT-1 transgene may not fully rescue *ept-1(0)* defects.
   1. *N-terminal GFP may partially compromise EPT-1 enzymatic activity, either directly or by partially impairing protein stability and/or localization. Alternatively, the extrachromosomal rescue may not be expressed at endogenous levels and is either underexpressed or overexpressed compared to controls.*

*Figure 4.* Is EPT-1 required for axon regeneration or axon maintenance during aging?

1. How comparable are *ept-1(0)* axon phenotypes to *cept-2(0)* phenotypes for regeneration and aging? If there are differences, what lipid-based reasons could explain this?
   1. *The defect in regeneration (~75% length of control) is comparable between ept-1 and cept-2 (Fig. 4a vs Fig. 1d). However, the mutants have different day 5 aging phenotypes: cept-2 causes a 100% penetrant PLM beading phenotype (Fig. 2a), whereas ept-1 causes degeneration in the middle/distal of the axon in 20% of mutants (Fig. 4b). As CEPT-2 can produce both PC and PE, but EPT-1 only produces PE, it implies that while PE is essential for regeneration, loss of PC biosynthesis may be the root cause of the beading phenotype in PLM axons.*
2. Why did the authors want to test *ept-1(0);cept-2(0)* double mutants? Did they?
   1. *The double mutants were lethal, preventing this epistasis experiment. If they weren’t, this would have been useful for exploring whether ept-1(0) and cept-2(0) were impacting the same lipid and cell biological processes (if regeneration in the ept-1(0);cept-2(0) double mutant was no worse than single mutant phenotypes, this implicates a shared biological process in regeneration; e.g., PE production).*
3. While the *ept-1* transgenes rescued proximal axon regrowth after axotomy, the authors noted that the terminal morphology of the neuron remained aberrant. What does it suggest about the different areas of axons, and the *ept-1* rescue constructs?
   1. *Axon terminals may be more sensitive to dosages of EPT-1, and thus to precise levels of PE biosynthesis, that are not produced by extrachromosomal EPT-1.*

*Figure 5*. What is the role of DIP-2 in axon regrowth?

1. What do the single and double mutants (*dip-2(0);cept-2(0)* and *ept-1(ju1698)dip2(0)*) suggest about the function of DIP-2? Does DIP-2 function depend on PE biosynthesis?
   1. *Single dip-2 and double mutants (dip-2(0) + ept-1(0) or cept-2(0)) all have increased regenerative capacity. In genetics parlance, this means the dip-2 phenotype (overgrowth) is epistatic to the ept-1 and cept-2 phenotypes (undergrowth). Moreover, it implies that DIP-2 is a brake on axon growth that does not depend on the individual function of EPT-1 or CEPT-2 and is likely independent of PE production via EPT-1*.
2. Which condition had the most prominent mean axon regrowth (Fig. 5a-b)? What might this suggest about DIP-2 and DAG function in axon regrowth?
   1. *dip-2(0);cept-2(0) double mutants. DAG levels are elevated in dip-2 mutations, but DAG could be converted via CEPT-2 to PC. Thus, the double mutant may have even higher levels of DAG. Intriguingly, high DAG promotes regeneration in PLM sensory neurons (Alam et al. 2016). In dip-2(0);cept-2(0) double mutants, DAG itself, or another DAG-based lipid, could directly fuel regrowth. Alternatively, TAG generated by DAG could supply energy for axon regrowth, and/or phosphatidic acid that is indirectly generated from DAG could fuel PI production and phosphorylated inositol signaling molecules that promote growth.*
3. How might you test if the *dip-2* mediated regrowth was functional in the ALM circuit?
   1. *Behavioral touch assays: touch responses should be impaired following axotomy but recover in functionally regrown circuits such as dip-2(0) mutants.*
4. What other functions could lipids play in regrowth, outside of structural bilayer components such as PE/PC?
   1. *Energy: fueling mitochondria by beta-oxidation. And signaling, through DAG/PA or phosphorylated PIPs (i.e., PI3K/Akt signaling pathways).*
5. What phenotypes do authors notice occurring in the *dip2;cept-2* and *dip2;ept-1* double mutants? How do authors quantify these phenotypes?
   1. *Ectopic neurites and axonal branching. Authors quantify the % of ALMs with ectopic neurites and branching, and % of PLMs with axonal branching. Authors also quantify the ectopic neurite length (short >10um, medium 10-30um, long 30-60um, and very long >60um).*

**Reference:**

Alam T, Maruyama H, Li C, Pastuhov SI, Nix P, Bastiani M, Hisamoto N, Matsumoto K. 2016. Axotomy-induced HIF-serotonin signalling axis promotes axon regeneration in C. elegans. Nat Commun. 7(1):10388. doi:10.1038/ncomms10388.
